# Mini-Exon Genotyping of *Leishmania* Species in Khuzestan Province, Southwest Iran

**Published:** 2010-03

**Authors:** J Saki, AR Meamar, H Oormazdi, L Akhlaghi, S Maraghi, M Mohebali, S Khademvatan, E Razmjou

**Affiliations:** 1 Department of Medical Parasitology and Mycology, Faculty of Medicine, Iran University of Medical Sciences, Tehran, Iran; 2 Department of Medical Parasitology and Mycology, Faculty of Medicine, Ahvaz Jundishapur University of Medical Sciences, Ahvaz, Iran; 3 Department of Medical Parasitology and Mycology, School of Public Health, Tehran University of Medical Sciences, Tehran, Iran

**Keywords:** Cutaneous leishmaniasis, Mmini-exon, RFLP, Sequencing, Iran

## Abstract

**Background:**

Leishmaniasis is a protozoan disease cause by *Leishmania* genus. Anthroponotic and zoonotic cutaneous leishmaniasis are endemic in Iran. The aim of this study was to identify the causative agent of cutaneous leishmaniasis by mini-exon gene in five regions of Khuzestan Province, southwest of Iran.

**Methods:**

From 2007 to 2008 in this cross-sectional study, cutaneous samples were collected from patients referred to Health Centers and Hospitals of the Khuzestan Province for cutaneous leishmaniasis diagnosis and cultured in Novy-MacNeal-Nicolle (NNN) and RPMI 1640. The propagated promastigotes were harvested and *Leishmania* species of cutaneous leishmaniasis were identified by RFLP and DNA sequencing of the PCR generated fragments.

**Results:**

*L. major* and *L. tropica* were the causative agents of cutaneous leishmaniasis by predominantly of *L. major* species. The alignment of the mini-exon sequencing isolates with reported sequencing of *L. major* and *L. tropica* revealed 92%-99% identity.

**Conclusion:**

Our study showed that mini-exon PCR-RFLP was useful method to identify the causative species of cutaneous leishmaniasis.

## Introduction

Leishmaniasis has a variety of clinical manifestations ranging from self-curing cutaneous lesions to fatal visceral form that is caused by parasitic protozoan *Leishmania* genus. Leishmaniasis is endemic in many parts of the world and remains a serious public health problem. Two species of *Leishmania* are involved in cutaneous leishmaniasis (CL) in Iran. *L. tropica* and *L. major* are the causative agents of anthroponotic cutaneous leishmaniasis (ACL) and zoonotic cutaneous leishmaniasis (ZCL), respectively (1). Based on conducted studies, Khuzestan Province is one of the important endemic areas of CL in Iran (2–5). In Iran–Iraq war lasting from September 1980 to August 1988, thousands of ZCL cases appeared among soldiers and paramilitary men who were sent to the war front in the south-west in the first two years (6). Correct identification of the *Leishmania* species is important for appropriate species-specific therapeutic as well as epidemiologic and genetic studies (7).

In the past decade, DNA-based methods have been used for diagnosis and identification of *Leishmania* species (8–11). Detection of the *Leishmania* parasites by PCR methods is highly sensitive and specific (12). Identifying *Leishmania* isolates using kinetoplast sequences as a target revealed extensive intraspecific polymorphism among strains of one species hence this method is less reliable (13). Among targets applied in DNA-based methods, mini-exon is highly specific and sensitive because the gene is present in all Kinetoplastida, whereas absent from the vertebrate host or invertebrate vector. Moreover, different *Leishmania* species have distinct length of the non-transcribed intergenic spacer region (14, 15).

Prior to this work, the majority of DNA-based molecular studies in Iran used internal transcribed spacer (ITS) in the ribosomal operons and kinetoplast DNA, such as minicircle sequences (2, 3) (16–22). To our knowledge, there have been no studies based on mini-exon gene in Iran. Consequently, we applied mini-exon PCR-RFLP and sequencing tools for identification of *Leishmania* species, in Khuzestan Province.

## Materials and Methods

### Samples collection

From August 2007 to May 2008 in a cross-sectional study, 128 cutaneous samples were collected from patients referred to Health Centers and Hospitals of the Khuzestan Province for cutaneous leishmaniasis diagnosis. The patients had given their informed consent for including their samples in our study. Geographically the Khuzestan Province is in the southwest of Iran, bordering Iraq's Basra Province, and the Persian Gulf. Its capital is Ahvaz and covers an area of 63,238 km^2^. The province has an estimated population of 4,277,998 inhabitants.

Five regions of the province were considered for sampling; North (Shush), South (Hendijan), West (Dashteazadegan: Susangerd, Hovizeh, Bostan), East (Ramhormoz) and Center (Ahvaz).

For each patient, a questionnaire including gender, age, patient location, and lesion type information was filled and then three samples were taken by scraping the internal border of skin lesions with a surgical blade.

Specimens were used for microscopical examination, culture in RPMI 1640 medium supplemented with 5% fetal bovine serum (FBS) and 200 IU/ml penicillin-streptomycin and culture in NNN (Novy-MacNeal-Nicolle) medium. Primary *Leishmania* isolates were subcultured in RPMI 1640 media with 10% FBS. Culture tubes were incubated at 25°C. The cultures were checked for promastigotes every 3 days for 4 weeks.

### DNA extraction

The mass cultured promastigotes were harvested by centrifugation (4000 rpm at 4°C for 15min) and washed three times in cold sterile PBS (pH 7.2). DNA was extracted by QIA DNeasy blood and tissue kit (QIAGEN, Hilden, Germany) according to the manufacturer's instructions. Purified DNA was eluted in 100 µl of elution buffer and stored in −20°C until use. PCR control DNA preparations were also extracted from *L. major* MHOM/IR/75/ER, *L. tropica* MHOM/IR/02/Mash 10 and *L. infantum* MCAN/IR/97/LON 49 (Iranian standard strains).

### PCR amplification

Amplification of the mini-exon gene was performed as a single PCR with forward (5'-TATTGGTATGCGAAACTTCCG-3') and reverse (5'-ACAGAAACTGATACTTATATAGCG-3') primers as described before (23). Two-seven µl of template DNA (75-100 ng/reaction) were amplified in 20 µl of modified Taq DNA polymerase Master Mix RED reaction (Bioneer Korea) containing 75 mM Tris-HCl (PH 8.5), 20 mM (NH _4_)_2_SO_4_, 1.5 mM MgCl_2_, 0.1% Tween20, 0.2 mM dNTPs, 0.25 unit Amplicon Taq DNA polymerase, 12% DMSO (78.13 g/mol Cinnagen Iran), 10 pM of each primer, inert red dye and a stabilizer.

DNA was amplified using thermal cycler (Eppendorf AG 22331, Hamburg, Germany) under the following conditions: 5 min at 94°C followed by 35 cycles of 30 sec at 94°C, 30 sec at 51.5°C, 45 sec at 72°C and a final elongation at 72°C for 10 min. For each sample, one positive control and one negative control were included.

The PCR products were separated on a 1.5% (W/V) agarose gel and visualized by staining with ethidium bromide.

### RFLP and sequencing

Fifteen µl of the PCR products were digested with 1.5 µl of *Eae* I (Fermentase life science, Germany) or 1.5 µl of *Hae* III (Takara Bio Inc, Japan) at 37°C for 2h (23). Digestion products were separated by gel electrophoresis in 2.5% agarose and visualized with ethidium bromide staining.Three Iranian reference strains and randomly 14 of 60 samples from five different parts of the province were selected based on the RFLP results. The PCR products of 17 samples were purified using an Accuprep Gel Purification kit (Bioneer, Deajeon, Korea) then sequenced (MWG-Biotech, Ebersberg, Germany) by the primers employed in the PCR.

Sequence alignments were constructed using the program CLUSTAL W version 1.83 (http://www.ddbj.nig.ac.jp/search/clustalw-e.html).

## Results

One hundred twenty eight patients with wet and dry skin lesions were enrolled in the study; 58 were male and 70 female. The median age was 11.25 years (range, 3 months to 75 years).

Only 60 (47%) culture tubes grew promastigotes after incubation at 25°C.

**Fig. 1:**
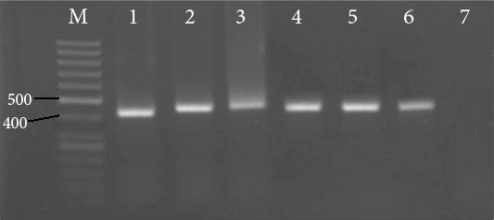
PCR products of Iranian reference strains and samples on 1.5% agarose gel. M: 50 bp molecular weight marker; lane 1: *Leishmania* *tropica* (MHOM/IR/02/Mash10); lane 2: *L. major* (MHOM/IR/75/ER); lane 3: *L. infantum* (MCAN/IR/97/LON 49); lanes 4-6: samples; lane 7: negative control (Distilled water instead of DNA template).

The PCR products of *L. tropica*, *L. major* and *L. infantum* reference strains and samples were around 410–440 bp (Fig. 1).

The PCR products of 60 isolates and 3 Iranian reference strains were purified and digested by *Eae* I restriction enzyme.

**Fig. 2:**
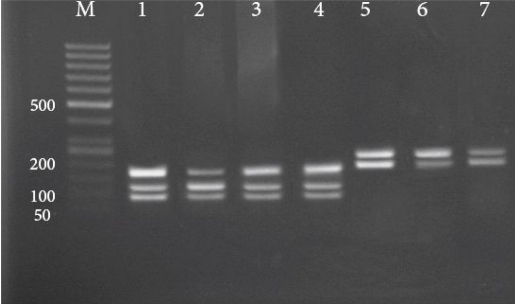
RFLP patterns of *Leishmania* species after digestion with *Eae* I (for *L. major*) and *Hae* III (for *L. tropica*) on 2.5% agarose gel. M: 50 bp molecular weight marker; lane 1: *L. major* (reference HOM/IR/75/ER); lane 2: *L. major* (center region); lane 3: *L. major* (south region); lane 4: *L. major* (west region); lane 5: *L. tropica* (reference MHOM /IR/02/Mash10); lane 6: *L. tropica* (center region); lane 7: *L. tropica* (west region)

RFLP analysis of most *L. major* samples and of *L. major* reference strain showed DNA fragments of 175, 120 and 94 bp (Fig. 2). The purified PCR products of both *L. tropica* samples and of the *L. tropica* reference strain however were failed to digest by *Eae* I restriction enzyme, even after overnight incubation at 37°C. Subsequently, these samples were digested using *Hae* III restriction enzyme and produced two fragments around 230 and 180 bp after incubation for 2h at 37°C (Fig. 2). Totally, based on RFLP profiles two different *Leishmania* species, *L. major* (96.6%) and *L. tropica* (3.4%), were identified from 60 samples. The two *L. tropica* species were belonged to Ahvaz and Dashteazadegan cities. To confirm the RFLP results, the amplified mini-exon gene of 14 isolates and 3 Iranian reference strains were sequenced. The results were submitted to DDBJ/Genbank at accession nos. **AB470338**, **AB470339** and **AB465575** for Iranian reference strains of *L. tropica*, *L. infantum* and *L. major,* respectively and **AB494686**-**AB494697** (*L. major*) and **AB494698**-**AB494699** (*L. tropica*) for the samples.

**Fig. 3:**
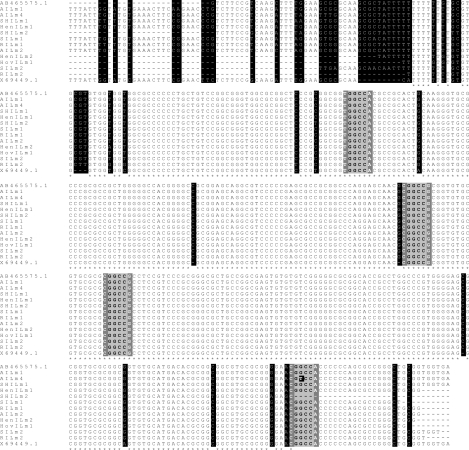
Multiple alignments of nucleotide sequences of mini-exon gene from 12 isolates, Iranian *L. major* reference strain (**AB465575.1**) and Israeli isolate (**X69449.1**). *L. major* isolates sequences are AILm1: **AB494695**; AILm4: **AB494690**; SHILm1: **AB494693**; HenILm1: **AB494688**; SHILm2: **AB494696**; SILm1: **AB494686**; RILm1: **AB494687**; AILm2: **AB494689**; HenILm2: **AB494692**; HovILm1: **AB494694**; SILm2: **AB494697**; RILm2: **AB494691**. Dashes indicate computer-generated gaps. Asterisks indicate identical nucleotides. Substitutions are shaded in black and the *Eae* I restriction sites are highlighted by gray boxes.

**Fig. 4:**
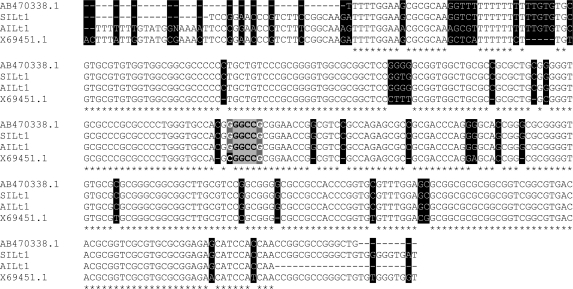
Multiple alignments of nucleotide sequences of mini-exon gene from 2 isolates, Iranian *L. tropica* reference strain (**AB470338.1**) and Sudanese isolate (**X69451.1**). *L. tropica* isolates sequences are SILt1: **AB494699**; AILt1: **AB494698**. Dashes indicate computer-generated gaps. Asterisks indicate identical nucleotides. Substitutions are shaded in black and the *Eae* I recognition site (5'-Py▼GGCCPu-3') are highlighted by gray boxes, dark gray boxes show Pyrimidine (C, T and U) and Purine (A and G) nucleotides where Guanine substitution instead of Cytosine in Iranian *L. tropica* are tinted with black.

The nucleotide sequences of mini-exon gene from isolates were aligned with nucleotide sequences of *L. major* (GenBank accession nos. **X69449.1** and **AB465575**) (Fig. 3) and *L. tropica* (GenBank accession Nos. **X69451.1** and **AB470338**) (Fig. 4). The Fig. 3 showed that *L. major*, which isolated from samples showed 2 to 8 (97%-99% homology) and 13 to 35 (92%-97% homology) nucleotide substitutions as compared to Iranian reference strain and Israeli isolate (**X69449.1**), respectively (Fig. 3). Furthermore, the Fig. 4 showed comparison substituted nucleotides of two *L. tropica* isolated with Iranian reference strain (4 and 6) by 97% and 98% identity and Sudanese isolate (**AB470338**)(34 and 46) by 88% and 91% homology.

## Discussion

Morphological discrimination of *Leishmania* species is not usually possible. The isoenzyme electrophoresis method requires cultured promastigotes but culture is time consuming, labor intensive and its sensitivity is suboptimal, as shown in our own study where only 47% of strains cultured grew promastigotes (24, 25). Over the last few years, several DNA-based molecular assays have been developed for species identification (26–28).

In the present study, we report the first application of mini-exon PCR and sequencing in order to characterize the *Leishmania* species causing cutaneous leishmaniasis in Khuzestan Province of Iran. The results showed that both *L. major* and *L. tropica* were occurring in this endemic area*, L. major* being by far the most prevalent. These findings are in agreement with previous studies. Maraghi *et al*. showed that 90% of cutaneous leishmaniasis isolates were *L. major* and the remaining were *L. tropica* (2), in Tashkori *et al.* (3) and Moatazadian *et al*. (4) studies, all cutaneous leishmaniasis isolates from Khuzestan Province were identified as *L. major*.

The mini-exon amplicon fragments yielded by both *L. tropica* and *L. major* were of 410-440 pb. Our findings are similar to those reported by Fernandes *et al.* on old world dermotropic and viscerotropic *Leishmania* species (14). Hence, mini-exon PCR is not discriminatory enough and for an accurate characterization, additional methods are needed. Our results suggest that mini-exon PCR-RFLP and sequencing to be very adequate for this purpose.

According to Manfurt *et al*., it is expected that all *Leishmania* species of the subgenus *Leishmania* can be distinguished by mini-exon PCR-RFLP, with *Eae* I being the most informative restriction enzyme (29). However, in both *L. tropica* Iranian samples and the reference *L. tropica* strain used in our study, *Eae* I failed to cut the mini-exon PCR products. The sequencing analyses revealed that our observation was due to one nucleotide substitution exactly in the *Eae* I recognition site. The nucleotide sequences alignment of Iranian *L. tropica* reference and isolates with Sudanica *L. tropica* isolate demonstrated Guanine (G) substitution instead of Cytosine (C) nucleotide in Iranian *L. tropica* causing this ineffectiveness of *Eae* I (Fig. 4).

Multiple alignments of mini-exon gene nucleotide sequences from isolates, Iranian reference strain and other sequences reported in GenBank revealed some nucleotides substitutions (Fig. 3 and 4). Despite these variations in mini-exon gene, *Eae* I restriction enzyme RFLP scheme in Iranian *L. major* reference and isolates were identical to those reported by Manfurt et al. (29) except in one isolate (**AB494690**) that substitution in restriction site altered the number of the fragments generated.

Therefore, in order to obtain unequivocal RFLP results, digestion of PCR products with *Hae* III along with *Eae* I is recommended.

In conclusion, our finding confirms that *L. major* is the predominant causative agent of cutaneous leishmaniasis in Khuzestan Province and mini-exon PCR-RFLP is a very practical method to identify the causative species of cutaneous leishmaniasis; and would therefore be very useful for identification of *Leishmania* in both vectors and reservoir hosts for further epidemiological studies in this endemic region.
